# Complete Genome Sequence of *Magnetospirillum gryphiswaldense* MSR-1

**DOI:** 10.1128/genomeA.00171-14

**Published:** 2014-03-13

**Authors:** Xu Wang, Qing Wang, Weijia Zhang, Yinjia Wang, Li Li, Tong Wen, Tongwei Zhang, Yang Zhang, Jun Xu, Junying Hu, Shuqi Li, Lingzi Liu, Jinxin Liu, Wei Jiang, Jiesheng Tian, Ying Li, Dirk Schüler, Lei Wang, Jilun Li

**Affiliations:** a State Key Laboratories for Agrobiotechnology and College of Biological Sciences, China Agricultural University, Beijing, China; b Tianjin Biochip Corporation, Tianjin, China; c Ludwig-Maximilian University Munich, Department Biology I, Microbiology, Planegg-Martinsried, Germany; d France-China Bio-Mineralization and Nano-Structure Laboratory, Beijing, China

## Abstract

We report the complete genomic sequence of *Magnetospirillum gryphiswaldense* MSR-1 (DSM 6361), a type strain of the genus *Magnetospirillum* belonging to the *Alphaproteobacteria*. Compared to the reported draft sequence, extensive rearrangements and differences were found, indicating high genomic flexibility and “domestication” by accelerated evolution of the strain upon repeated passaging.

## GENOME ANNOUNCEMENT

*Magnetospirillum gryphiswaldense* MSR-1 was first isolated in the mud of the little eutrophic Ryck River near Greifswald, Germany, by D. Schüler and was described by Schleifer et al. in 1991 (1). At the time, it represented one of the first magnetotactic bacterium strains available in axenic lab culture.

The full genome sequence was assembled from Illumina Solexa and Roche 454 reads. Gene annotation and genome analysis were performed by the MaGe Genoscope platform service (2), by which manual annotations of all predicted genes were completed. The genome consists of a single circular chromosome with 4,365,796 bp. The average G+C content is 63.28%. The chromosome contains 4,261 coding sequences (CDSs), 50 tRNAs, 2 sets of rRNA, and 11 miscellaneous RNAs (misc_RNAs). The average CDS length is 954.81 bp.

Automatic classification of clusters of orthologous groups (COG) of proteins showed that 78.01% of CDSs (3,324 out of 4,261) were classified in at least one COG group (3). Proteins belonging to signal transduction mechanisms, amino acid transport and metabolism, and inorganic ion transport and metabolism correspond to 11.95%, 10.68%, and 8.71% of the gene products that can be classed in COG groups. The percentage of open reading frames (ORFs) of unknown functions (about 37%) is the same as the percentage of enzyme-coding genes (including putative enzymes), which make up more than one-third of the total ORF coding products. A total of 1,924 of the predicted gene products of all 4,261 MaGe CDSs have unknown cellular localization.

The genomic segment comprising several gene clusters (*mms6, mamFDC, mamAB*, and *mamXY*, for a total of 27 genes; 23.6 kb) that encode the majority of magnetosome proteins shares many of the hallmarks of genomic islands often associated with pathogenic organisms (4). Magnetosome island (MAI) boundaries were identified from MGMSRv2_2285 to MGMSRv2_2412 (about 102 kb) by a method for gene island determination (5). The G+C percentage of the MAI is 60.99%, and 33 genes were annotated as transposases, implying that the MAI is instable. The *mam* genes were checked and it was found that *mamG* was not present. Compared to the draft genome sequence, small-scale mismatches were found in other *mam* genes, like *mamC* and *mamJ*.

The first (and perhaps most critical) step in magnetite biomineralization is the transport of iron from the extracellular environment into the cell. Although no siderophores were experimentally detected in MSR-1 (6), one gene coding for a putative ferrous siderophore (MGMSRv2_3314) was found. An ABC-TonB-ExbBD system represents a ferric iron transport system (7). MSR-1 has a complete TonB and ExbBD system. Four TonB-dependent receptors, two TonB proteins, two TonB C-terminal domains containing proteins, and dozens of ABC transporters, including a ferric iron ABC transporter, are encoded in the genome. A ferrous iron transport system (*feo*) is related to the ferrous transport system of other bacteria, and two *feo* operons are present in MSR-1 (8). One Fe(III) reductase-coding gene (MGMSRv2_0005) was identiﬁed, and five ferric uptake regulator-like genes belong to the *fur* family.

### Nucleotide sequence accession number.

This whole-genome project has been deposited in DDBJ/EMBL/GenBank under the accession no. HG794546. The assembled sequences and annotations are available in the MicroScope Microbial Genome Annotation & Analysis Platform at https://www.genoscope.cns.fr/agc/microscope/home/index.php.
